# Augmented Reality in Orthopedics: Current State and Future Directions

**DOI:** 10.3389/fsurg.2019.00038

**Published:** 2019-06-27

**Authors:** Dimitrios Chytas, Michael-Alexander Malahias, Vasileios S. Nikolaou

**Affiliations:** ^1^2nd Orthopaedic Department, School of Medicine, National and Kapodistrian University of Athens, Athens, Greece; ^2^Complex Joint Reconstruction Center, Hospital for Special Surgery, New York, NY, United States

**Keywords:** augmented reality, orthopedics, perspective, future, current status

## Abstract

Augmented reality (AR) comprises special hardware and software, which is used in order to offer computer-processed imaging data to the surgeon in real time, so that real-life objects are combined with computer-generated images. AR technology has recently gained increasing interest in the surgical practice. Preclinical research has provided substantial evidence that AR might be a useful tool for intra-operative guidance and decision-making. AR has been applied to a wide spectrum of orthopedic procedures, such as tumor resection, fracture fixation, arthroscopy, and component's alignment in total joint arthroplasty. The present study aimed to summarize the current state of the application of AR in orthopedics, in preclinical and clinical level, providing future directions and perspectives concerning potential further benefits from this technology.

## Introduction

Computer-assisted orthopedic surgery has gained increasing interest, especially in the last two decades (1), being employed for surgical planning, simulation, and navigation (2). Via navigation, which is the core element of computer-assisted orthopedic surgery systems, orthopedic surgeons could accurately track and intuitively visualize surgical instruments in real time, in relationship with anatomical structures (1). The human-machine interface, an integral part of image-guided orthopedic navigation systems, provides a platform to merge preoperative and intraoperative images from different modalities and three-dimensional models to facilitate operative planning and navigation (3). The rapid development of augmented reality (AR) technologies has the potential to lead to an ideal form of human-machine interface (3).

AR has been described as “the concept of digitally superimposing virtual objects onto physical objects in real space so individuals can interact with both at the same time” (4). In contrast to AR, in virtual reality (VR) the whole simulation occurs exclusively in a computer-generated environment (5). An AR system comprises special hardware and software, which is used in order to offer computer-processed imaging data to the surgeon in real time, so that real-life objects are combined with computer-generated images (5). Specifically, computer-generated images are superimposed to real-world images and are displayed via a video projector, computer or tablet (6–12).

**Figure 1:**
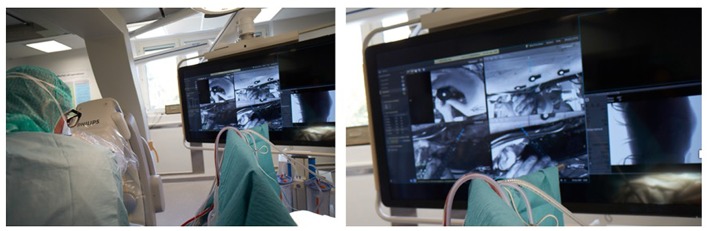
**(Left)** Picture showing a surgeon from Karolinska hospital in Stockholm using a hybrid operating room with an augmented reality system integrated with the robotic C-arm. **(Right)** The surgeon is seeing at the screen the video streaming of his surgery with a blue line indicating the direction of his instruments that he is navigating into the pedicle (Photo courtesy of Dr. Rami Nachabe).

The basic structure of an AR system used in orthopedic surgery, as it was described by Nikou et al. (13), comprises three essential elements: a position tracking system, a display device and a system control software (14). The position tracking system monitors the location and orientation of the objects in the operative field. Medical imaging techniques, such as fluoroscopic images, are used as part of the tracking system. Markers, such as metal spheres, visible by the imaging modality, are attached to the patient or to surgical tools and contribute to the determination of the relative position of the objects in the operative field. The system control software uses the information from the tracking system and transforms the input into images, which are sent to the display system, where the combination with the view of the real scene takes place (13, 15, 16). The display system could be head-mounted (5) (Figure 1).

**Figure 2:**
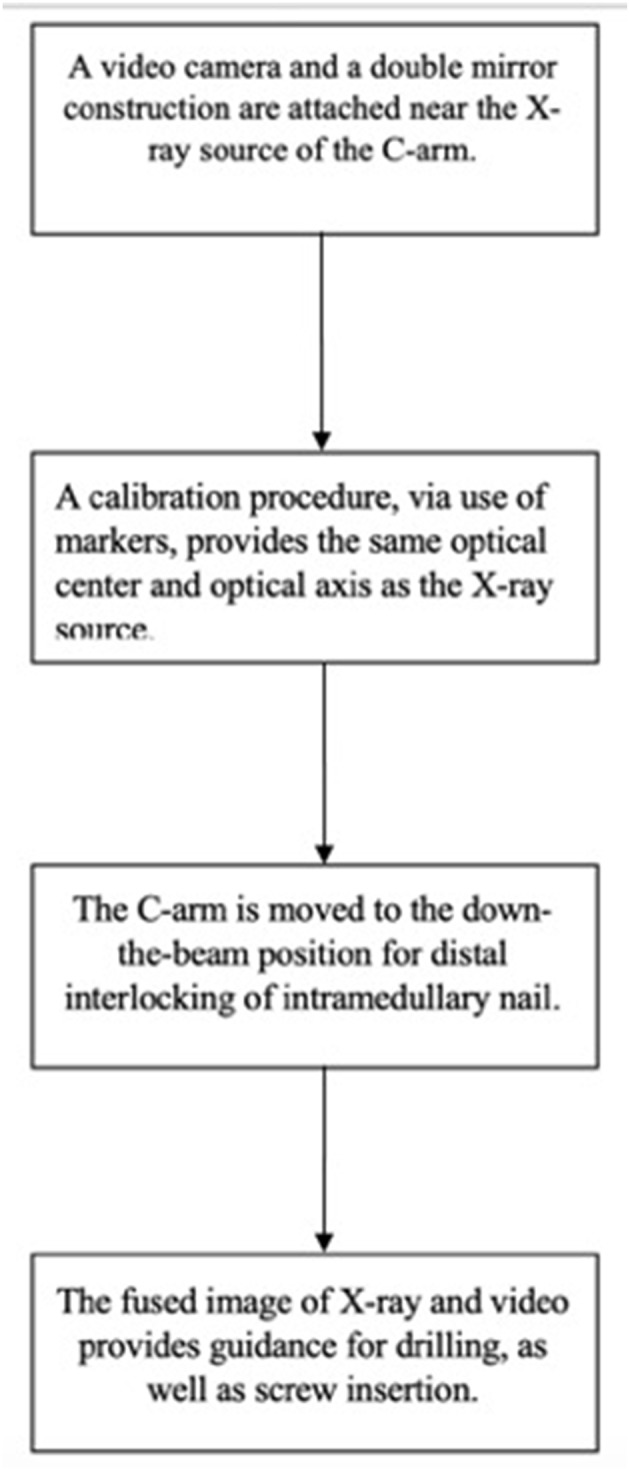
A diagram which depicts the use of a camera-augmented C-arm for distal interlocking of an intramedullary nail.

**Figure 3:**
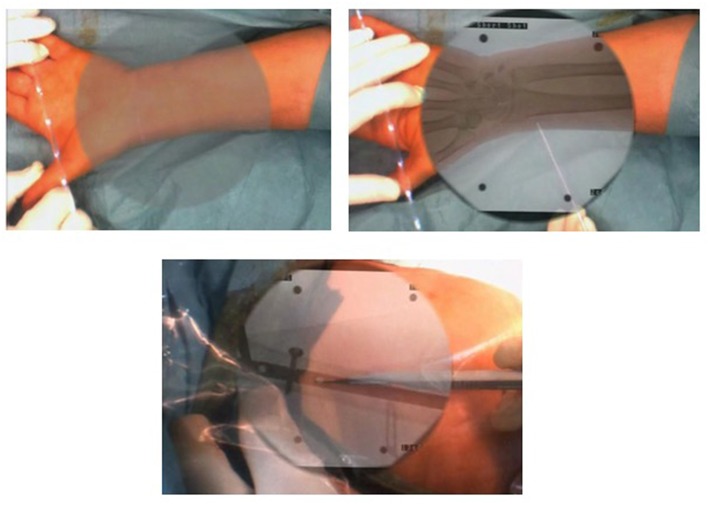
Examples of intraoperative image in wrist surgery and distal locking intramedullary nailing (Photos courtesy of Prof. Pascal Fallavolitta, Ottava, Canada).

There is a wide variety of AR systems which have been implemented in preclinical and clinical level. An example of a camera-augmented C-arm was described by Navab et al. (15). A video camera and a double mirror system are placed near the X-ray source of the C-arm, so that the optical center of the camera virtually coincides with the X-ray source. Markers, which are attached during the calibration of the system and are simultaneously visible by both X-ray source and video camera, lead to a valid overlay of X-ray and video image. Thus, the surgeon's vision is concentrated in a single view, which is a significant advantage of AR in comparison with other visualization techniques (5). A diagram of the procedure of the use of this type of C-arm in a case of distal interlocking of an intramedullary nail (15) is depicted in Figure 2. Examples of intraoperative image in wrist surgery and distal locking in intramedullary nailing are demonstrated in Figure 3.

Based on preclinical data, Blackwell et al. (14) remarked that AR could possibly be applied to a wide spectrum of orthopedic procedures, such as tumor resection, fracture fixation and component's alignment in total joint arthroplasty. Since then, the implementation of AR in both laboratory and clinics has been accompanied by encouraging results, which indicate that AR could be proved a valuable tool for intra-operative guidance and decision-making.

We aimed to investigate the current state of the implementation of AR in orthopedic surgery. We searched in PubMed and Cochrane Database of Systematic Reviews, using the key words: “AUGMENTED REALITY” AND (“ORTHOPAEDIC” OR “ORTHOPAEDICS” OR “ORTHOPEDIC” OR “ORTHOPEDICS” OR “ARTHROPLASTY” OR “REPLACEMENT” OR “ARTHROSCOPY” OR “FRACTURE” OR “SPINE”). Our inclusion criteria were: studies published after January 2010 and until September 2018 (end of our search), written in English language, with a purpose to investigate the outcomes of the implementation of AR in a specific orthopedic surgical intervention, either in preclinical or in clinical level. We excluded conference papers, expert opinions and review articles. In total, 91 studies were found after the initial search. Finally, 22 preclinical and nine clinical studies met our inclusion and exclusion criteria.

## Preclinical Studies About the Use of Augmented Reality in Orthopedics

Several preclinical studies have been conducted during the last two decades, which have demonstrated that AR could improve the accuracy of interventional procedures. Firstly, there has been substantial evidence published to support the use of AR in fracture surgery. Van Duren et al. (17) described the use of AR for simulating dynamic hip screw (DHS) insertion for the treatment of extracapsular hip fractures. The authors, who used a workshop bone, noted that AR could be proved useful in training orthopedic surgeons to perform DHS fixation, by offering to them an easily accessible, affordable and realistic simulation of guide-wire insertion (17). In addition, Hiranaka et al. (18) examined whether AR could improve the accuracy of K-wires' placement on plastic femurs, from the lateral cortex to the femoral head center. They noted that the AR system led to significantly decreased placement time, radiation exposure time and tip apex distance (and thus more accuracy), compared to the conventional method (18).

Londei et al. (19) illustrated that AR-guided distal locking during the placement of intramedullary nails (IMN) was a safe procedure which could reduce the operative time, while the accuracy of surgeons was ameliorated in comparison with other available methods of distal locking. Diotte et al. (20) used an AR fluoroscope to design a radiolucent drill, which permitted surgeons to perform distal locking of IMN to bone phantoms, using only a single X-ray image. AR navigation, according to Ma et al. (21), was also proved a method which offered satisfactory targeting accuracy of distal locking of IMN in a leg phantom and tibia models, while the authors pointed out reduced exposure of surgeons and patients to radiation. Similarly encouraging results were depicted by Unberath et al. (22), who found that AR-based C-arm positioning was accompanied by reduction of radiation dose, as well as by prevention of operator errors. Befrui et al. (23) showed that the number of fluoroscopic images, time and radiation dose were significantly reduced in comparison with X-ray imaging when AR technology was applied to navigated osteosynthesis of pelvic fractures in simulated bone structures. The use of AR-guided systems in non-traumatic cases of orthopedic surgery has also attracted several authors' interest. Cho et al. (24) explored the use of AR-based navigation in tumor resection in animal femurs and found that AR was accompanied by satisfactory accuracy, while there was not any additional cost or time spent. The positive outcomes concerning accuracy in tumors resection were also demonstrated in the study by Choi et al. (25). The authors experimented in animal pelvic bones to find that AR-based navigation led to significantly better results in comparison with the conventional methods, regarding the invasion of the target resection margin (25). Apart from the aforementioned studies which proved AR effectiveness concerning accuracy, Wang et al. (26) showed that AR led also to satisfactory precision in the percutaneous placement of sacroiliac screws.

Fallavollita et al. (27) assessed the use of AR-based C-arm technology to determine the alignment of lower limb in a cadaveric study. The authors depicted that AR led to valid measurements of the deviation of lower limb's mechanical axis, while only few X-ray image acquisitions were needed for the clinicians who participated in the study (27). In terms of total knee arthroplasty (TKA), Pokhrel et al. (28) proposed an AR-based system which might improve the accuracy of osteotomies, so that it might potentially improve the long-term outcomes of TKA. Logishetty et al. (29) evaluated the use of AR for training medical students in acetabular cup placement for total hip arthroplasty, in a phantom pelvis. The participants were found to be equally accurate when trained either by an expert surgeon or AR. As a result, the authors concluded that the latter method could be a valuable educational tool, since it was showed that some skills for arthroplasty can be learned without supervision (29). Fotouhi et al. (30) noted that AR technology could lead to limited radiation, simple and accurate placement of acetabular implant during total hip arthroplasty. Moreover, Liu et al. (31) explored the application of AR for hip resurfacing, using femur phantoms, and remarked that the system was accurate. Finally, Zemirline et al. (32) performed a preclinical study to investigate the implementation of AR for navigation during wrist arthroscopy. It was noted that the maneuver was made more intuitive, while, simultaneously, time was saved, concentration and comfort were enhanced (32).

Utilization of AR in spine surgery has been investigated by various researchers. Gibby et al. (33), who used a lumbar model, found promising results regarding the implementation of AR in surgical navigation for pedicle screw fixation, without real-time fluoroscopy. Elmi-Terander et al. (34) carried out a cadaveric study which comprised AR-assisted minimally-invasive placement of thoracolumbar pedicle screws. The authors concluded that navigation with AR is a method which can lead to effective and accurate minimally-invasive fixation, without radiographic control (34). In another study conducted by Elmi-Terander et al. (35), AR-assisted navigation without fluoroscopy was proved significantly superior to free-hand technique in terms of accuracy of thoracic pedicle screw fixation. Luciano et al. (36) found similar results in their study, in which thoracic pedicle screws were placed on a simulator of a patient's thoracic spine, constructed via a computed tomography of a real patient. Interestingly, the authors remarked a tendency toward learning retention, as well as a statistically significant improvement of the performance accuracy from practice to test sessions which took place (36). Also, Ma et al. (37) proposed an AR-based navigation system for pedicle screw placement, which was found to have satisfactory targeting accuracy and to limit radiation exposure to the patient and surgeon (37). Pedicle screw insertion was performed with accuracy in another preclinical study performed by Liang et al. (38).

## Clinical Studies About the Use of Augmented Reality in Orthopedics

Various clinical studies assessing the usefulness of AR in orthopedic surgery have documented satisfactory outcomes (Table 1). In the study by Von der Heide et al. (39), a camera-augmented C-arm system was compared with traditional C-arms in several procedures of plate, nail and screw osteosynthesis and implant removal. While the X-ray shots were diminished to approximately a half with the use of AR, the time of surgery remained similar to that of traditional C-arms (39). In a pilot study, Ponce et al. (40) investigated the role of an AR system in orthopedic residents' education as for shoulder arthroscopic procedures and it was noted that AR technology was a useful teaching tool, characterized by comparable operative times to conventional methods. Moreover, Ponce et al. (41) showed satisfactory range of motion and pain reduction in a patient who underwent total shoulder replacement via AR technology.

**Table 1:** Clinical studies about augmented reality in orthopedics.

**References**	**Field**	**AR system**	**Main outcomes**
Von der Heide et al. (39)	Several procedures performed with fluoroscopy (plate, nail and screw osteosynthesis, implant removal)	Camera-augmented C-arm	The X-ray shots were diminished to approximately a half with the use of AR. The time of surgery remained similar to that of traditional C-arms
Ponce et al. (40)	Shoulder arthroscopy	VIP	AR technology was a useful teaching tool for orthopedic residents, it was safe and was characterized by comparable operative times to conventional methods
Ponce et al. (41)	Total shoulder arthroplasty	VIP combined with wearable computing device	Satisfactory postoperative range of motion and pain reduction in a patient who underwent total shoulder arthroplasty
Ogawa et al. (42)	Total hip arthroplasty	AR-HIP	AR was significantly more accurate than the goniometer regarding the intraoperative measurement of the angles of acetabular cup fixation
Shen et al. (43)	Pelvic and acetabular fractures	A virtual fracture reduction system and an AR-aided templating system, comprising a personal computer and a video camera	AR-based reconstruction plate may lead to reduction of the operative time, surgical invasiveness and complexity
Elmi-Terander et al. (44)	Spine surgery	AR surgical navigation system, based on video input from four cameras mounted into the frame of a C-arm detector	AR-based surgical navigation could offer acceptable time of navigation and high accuracy of placement of pedicle screws
Wu et al. (2014)	Spine surgery	ARCASS	The AR-based system was characterized by feasibility, accuracy, reduced operative time and radiation dose to patients
Abe et al. (45)	Spine surgery	VIPAR	The AR-based system offered a remarkable help to surgeons to find the ideal needle trajectory and insertion point when performing percutaneous vertebroplasty
Kosterhon et al. (46)	Spine surgery	A system which preoperatively creates virtual resection planes and volumes for spinal osteotomies and exports three-dimensional operative plans to a navigation system controlling intraoperative visualization via a surgical microscope's head-up display	Increased accuracy and safety in a patient who underwent surgery for congenital hemivertebra of the thoracolumbar spine

*AR, augmented reality; VIP, Virtual Interactive Presence (merges in real time two video streams that capture separate and remote fields into a common task field, thus permitting real-time interaction between remote surgeons in that field); AR-HIP, a system which enables the surgeon to view an acetabular cup image superimposed on the real surgical field through the display of a smartphone, which shows anteversion angles and inclination of the acetabular cup; ARCASS, Augmented Reality Computer Assisted Spine Surgery (projects a preoperative three-dimensional model of the patient onto the intraoperative scene, using a camera and a projector); VIPAR, virtual protractor with augmented reality (comprises a head-mount display with a tracking camera and a marker sheet to visualize a needle trajectory in three-dimensional space during percutaneous vertebroplasty)*.

Ogawa et al. (42) evaluated the use of AR in total hip arthroplasty to demonstrate that AR was significantly more accurate than the goniometer regarding the intraoperative measurement of the angles of acetabular cup fixation. Shen et al. (43) developed an AR-based patient-specific reconstruction plate for pelvic and acetabular fractures to evaluate the clinical effectiveness of this treatment in a series of patients and found that the implant that they designed might lead to reduction of the operative time (43).

Several clinical studies were conducted regarding spine surgery. According to Elmi-Terander et al. (44), AR surgical navigation could offer high accuracy to the placement of thoracic and lumbosacral pedicle screws, while no screws were severely misplaced and no device-related complications were noticed. Wu et al. (45) evaluated the usefulness of AR technology in spine surgery and demonstrated the feasibility and accuracy of the AR system, while the surgeons who participated in the study noted reduced operative time and radiation dose. Kosterhon et al. (47) reported the use of AR in a patient with congenital hemivertebra of the thoracolumbar spine, and the system was found useful for the surgeon during the resection of a complex-shaped bone wedge, offering more accuracy and patient safety. Finally, Abe et al. (46) dealt with the value of AR technology in percutaneous vertebroplasty. The authors, who included a spinal phantom and patients in their study, concluded that the system offered a remarkable help to surgeons to find the ideal needle trajectory and insertion point (46).

## Future Directions-Conclusion

As it has been showed, the implementation of AR could possibly lead to improved accuracy in positioning and decreased radiation in a wide spectrum of orthopedic procedures, concerning either trauma or elective orthopedic surgery. However, the cost-effectiveness of AR in orthopedics is a factor which should be critically estimated (48) before the establishment of its routine use. Especially, a close interaction between scientists, clinicians and industry is essential (48), so as to be showed if AR systems can survive in a competitive environment (48) and if they are more cost-effective than other computer-assisted systems.

Also, concerns have been raised about the extent to which display of information via AR could be misleading or disturbing during surgery (49). Adequate contrast and clarity of the AR technology, as well as avoidance of masking structures in the real patient view, are of particular significance (49). Contrast could be enhanced via display of virtual data in strong primary colors (49). It is essential for the orthopedic surgeon to choose to use AR only when it is needed. This purpose could be achieved with functionality via a user interface that permits data models to be displayed or turned off (49). Moreover, adverse effects of the use of AR have been reported, especially dizziness (50). It remains to be explored to what extent such adverse effects will outweigh the benefits of the use of this technology in orthopedic surgery.

Although it has been shown that AR might have a beneficial impact to orthopedic residents' training, it would be interesting to investigate to what extent AR could play a significant role in providing orthopedic trainees with a valuable learning experience. Moreover, verifying the learning curve would be an interesting aim (42), which seems to need further research to be explored. A question to be answered is whether AR can substitute or enhance computer-navigated or computer-assisted navigation or robotic-assisted total joint arthroplasty. Since there is a growing interest in surgical variables that are intraoperatively controlled by orthopedic surgeons, including lower leg alignment, component positioning and soft tissues balancing and more tight control over these factors is associated with improved outcomes, several computer navigation and robotic-assisted systems have been developed (51). Although still controversial, the development of these techniques is among the most significant changes that might potentially improve patient outcomes (52). AR should be compared to these techniques as for the costs, operative time, accuracy, and clinical outcomes, since there is a lack of studies which include such comparison.

In addition, under conditions of real-time surgery, there are factors which might be critical and there are absent in preclinical studies. For example, the effect of patient's respiratory movement has to be taken into account during thoracic spine surgery (35). Moreover, it should be assessed if AR could effectively improve the accuracy of fixation of particular fractures or dislocations, involving, for example, the sacroiliac joint (26). Future studies could illustrate various points which remain to be explored. For instance, in terms of bone tumors, further research is needed to clarify if AR could achieve safer resection margins, less morbidity and better physical functioning of patients, in comparison with other techniques (48). Furthermore, it may be investigated if the application of the AR technology would be of benefit for cervical spine operations (46). Regarding DHS fixation, it would be interesting to be showed if AR could be helpful concerning reaming and screw insertion steps (17). As far as total hip arthroplasty is concerned, additional research is required to shed light on the potential of AR as a navigation tool (42).

We should note that the papers which were found after our search and met our inclusion and exclusion criteria might not cover the whole spectrum of the implementation of AR in orthopedic surgery and, also, could be soon outdated by the ongoing research. In summary, further clinical studies are required to confirm the favorable preclinical outcomes of the use of AR in orthopedics.

## Author Contributions

DC did the literature search and wrote the first draft of the manuscript. M-AM proofread the manuscript and made corrections. VN had the initial idea of writing the manuscript, proofread, and edited the manuscript.

### Conflict of Interest Statement

The authors declare that the research was conducted in the absence of any commercial or financial relationships that could be construed as a potential conflict of interest.
